# The Differential Effect of Arm Movements during Gait on the Forward Acceleration of the Centre of Mass in Children with Cerebral Palsy and Typically Developing Children

**DOI:** 10.3389/fnhum.2017.00096

**Published:** 2017-03-01

**Authors:** Pieter Meyns, Guy Molenaers, Jacques Duysens, Ilse Jonkers

**Affiliations:** ^1^Department of Rehabilitation Medicine, VU University Medical Center, Amsterdam Movement SciencesAmsterdam, Netherlands; ^2^Department of Kinesiology, Faculty of Kinesiology and Rehabilitation SciencesKU Leuven, Heverlee, Belgium; ^3^Clinical Motion Analysis Laboratory, CERM, University Hospital LeuvenLeuven, Belgium

**Keywords:** cerebral Palsy, upper limbs, walking, induced acceleration, propulsion

## Abstract

**Background:** We aimed to study the contribution of upper limb movements to propulsion during walking in typically developing (TD) children (*n* = 5) and children with hemiplegic and diplegic cerebral palsy (CP; *n* = 5 and *n* = 4, respectively).

**Methods:** Using integrated three-dimensional motion capture data and a scaled generic musculoskeletal model that included upper limbs, we generated torque driven simulations of gait in OpenSim. Induced acceleration analyses were then used to determine the contributions of the individual actuators located at the relevant degrees of freedoms of the upper and lower limb joints to the forward acceleration of the COM at each time point of the gait simulation. The mean values of the contribution of the actuators of upper limbs, lower limbs, and gravity in different phases of the gait cycle were compared between the three groups.

**Findings:** The results indicated a limited contribution of the upper limb actuators to COM forward acceleration compared to the contribution of lower limbs and gravity, in the three groups. In diplegic CP, the contribution of the upper limbs seemed larger compared to TD during the preswing and swing phases of gait. In hemiplegic CP, the unaffected arm seemed to contribute more to COM deceleration during (pre)swing, while the affected side contributed to COM acceleration.

**Interpretation:** These findings suggest that in the presence of lower limb dysfunction, the contribution of the upper limbs to forward propulsion is altered, although they remain negligible compared to the lower limbs and gravity.

## Introduction

Arm swinging during walking has been proposed to have several benefits in healthy adults (Meyns et al., 2013), including optimization of energy consumption (Collins et al., 2009) and reduction of instability (Ortega et al., 2008). Given the positive effect of arm swing in healthy subjects, a significant assistive effect is to be expected in patient populations. In previous studies, we found that children with diplegic (DiCP) and hemiplegic (HeCP) Cerebral Palsy (CP) adopt specific arm postures related to their gait instability (Meyns et al., 2012a). Additionally, HeCP are known to walk with a decreased arm swing on the hemiplegic side due to the altered tone of the muscles in their hemiplegic arm (Meyns et al., 2011). Contrarily, their arm swing on the non-hemiplegic side is significantly increased, which was found to counteract an increased angular momentum produced by the legs. As such, the non-hemiplegic arm aims to control total body angular momentum (Bruijn et al., 2011). Furthermore, it is suggested that DiCP are able to increase walking speed more than HeCP (despite the two affected lower limbs in DiCP) due to more adequate compensations of both (unaffected) upper limbs (Meyns et al., 2011).

Although the arms were found not to make a significant contribution to the forward acceleration of the center of mass (COM) in healthy subjects (Hamner et al., 2010; Hamner and Delp, 2013), it can be questioned whether in CP patients the arm movements contribute more to gait propulsion. This is particularly relevant as arm movements play a more important role for locomotion in CP compared to typically developing (TD) children. Therefore, we expected that the arms' contribution to propulsion was different between HeCP, DiCP, and TD. Specifically, we hypothesized that the contribution to propulsion of the COM was increased for both arms in DiCP and for the unaffected arm in HeCP compared to TD.

## Materials and methods

Five TD and nine CP children (five HeCP and four DiCP) participated (see Table 1).

**Table 1:** **Patient characteristics**.

	**TD**	**DiCP**	**HeCP**
*N*	5	4	5
Trials	8	6	7
Gender (M/F)	2/3	3/1	5/0
GMFCS (I/II)	–	3/1	4/1
Age (years)	8.40 ± 1.50	10.50 ±1.66	9.00 ± 2.28
Weight (kg)	32.54 ± 8.37	32.98 ± 6.26	28.70 ±7.09
Height (cm)	136.96 ± 10.36	142.93 ± 12.83	132.46 ± 9.90

*Table 1 presents characteristics of the three groups (Typically Developing children [TD], children with diplegic cerebral palsy [DiCP], and children with hemiplegic cerebral palsy [HeCP])*.

*Note that age, weight and height are presented as follows: Mean ± standard deviation. N, number of subjects; M/F, male/female; GMFCS, Gross Motor Function Classification System*.

CP children were recruited from the Clinical Movement Analysis Laboratory at UZ Pellenberg (UZ Leuven). They were ambulant (without walking aids), were diagnosed with the predominantly spastic type of CP, and had sufficient cooperation to follow verbal instructions. They did not receive lower limb surgery or did not undergo Botulinum Toxin treatment within the past 6 months.

The local ethical committee (Commissie Medische Ethiek KU Leuven) approved the experiments (approval number S51498). In accordance with the Declaration of Helsinki, written informed consent was obtained of the participants' parents.

The total-body PlugInGait marker set was used to collect three-dimensional kinematic data with an eight camera Vicon system (Oxford Metrics, Oxford, UK) at 100 Hz (see also Meyns et al., 2011). Marker coordinates were filtered and smoothed using Woltring's quintic spline routine. Workstation (5.2beta 20, Oxford Metrics) was used to define gait cycles and label individual marker trajectories. Ground reaction forces were measured using two force plates (AMTI, Watertown, MA) embedded in the 10 m walkway. Kinematic and kinetic data is publicly available on the SimTK website for other researchers to evaluate and use for future research (https://simtk.org/projects/cp-child-gait). All participants walked barefoot at self-selected walking speeds. Since in HeCP, one side of the body is significantly more affected, both sides were investigated separately: The most affected side was defined as the side which showed the highest median spasticity score on the Modified Ashworth Scale. In DiCP and TD data on both sides of the body were averaged.

**Figure 1:**
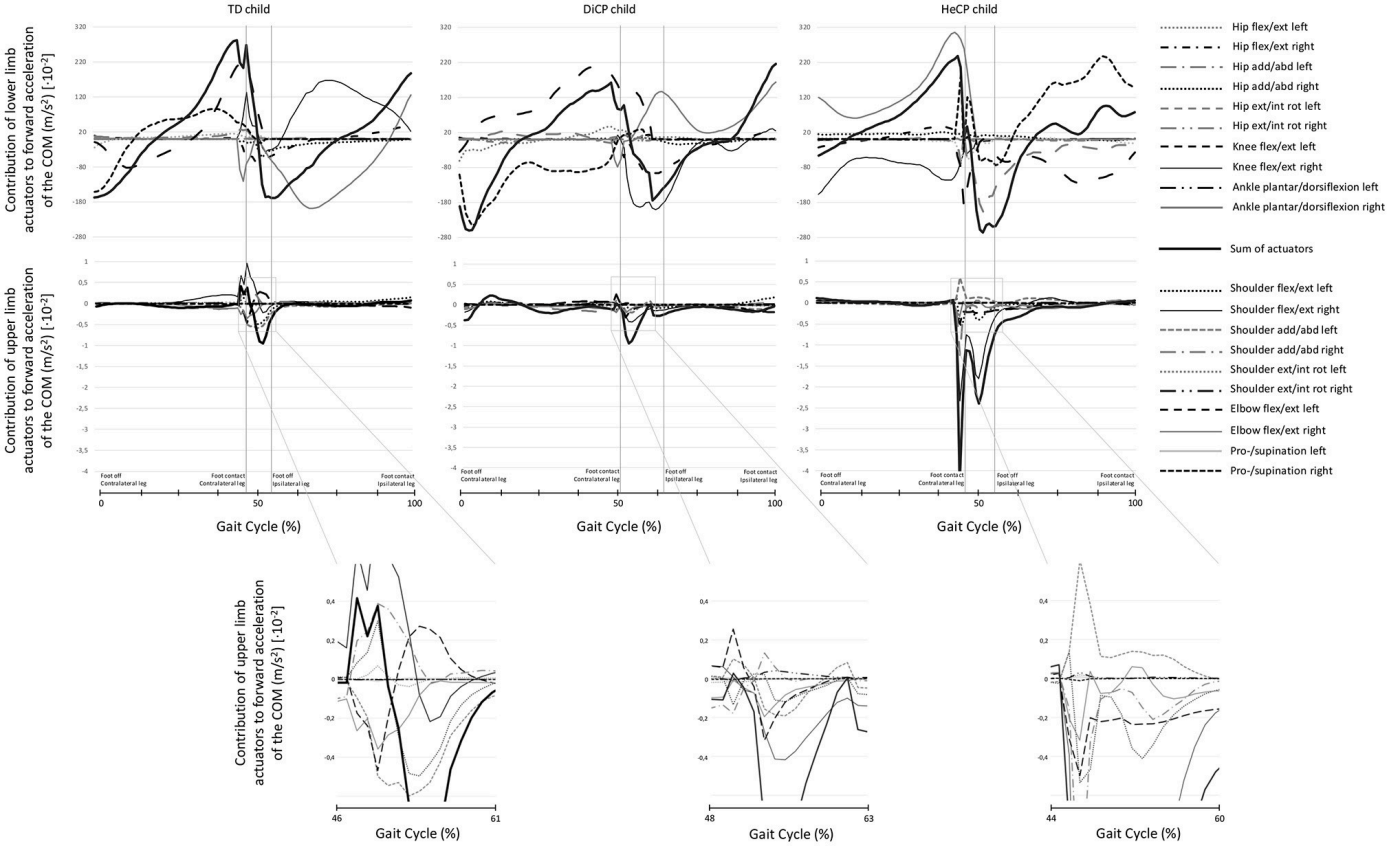
**Examples of individual data of the induced acceleration analysis representative for the group**. The contribution to forward acceleration/deceleration of the COM in three individuals (a Typically Developing child [TD; left column], a child with diplegic cerebral palsy [DiCP; middle column], and a child with hemiplegic cerebral palsy [HeCP; right column]) for the lower limbs (upper row) and upper limbs (lower row) are presented over the gait cycle. The sum of the contribution of the different actuators (thin lines) of the lower limbs is presented as a thick black line (upper row). The actuators of the lower limbs included (left and right); hip extension/flexion, hip abduction/adduction, hip external/internal rotation, knee flexion/extension, ankle dorsiflexion/plantarflexion. Similarly, the sum of the contribution of the different actuators (thin lines) of the upper limbs is presented as a thick black line (lower row). The actuators of the upper limbs included (left and right); shoulder extension/flexion, shoulder abduction/adduction, shoulder external/internal rotation, elbow flexion/extension, pronation/supination. To increase readability of the graphs, for the actuators of the upper limbs a part of the graph is enlarged close to preswing. As such, the contribution of the different upper limb actuators to forward acceleration of the COM can be better distinguished for each participant. Vertical lines indicate foot contact and foot off gait events.

Based on the integrated 3D motion capture data, dynamic torque-driven simulations of gait were created in OpenSim (Delp et al., 2007), using a model with 14 segments and 21 degrees of freedom (including three shoulder motion DOFs and one elbow motion DOF). This model was scaled to the anthropometry of the individual based on the marker positions measured during a static trial and body weight. Inverse kinematics were used to compute joint angles for the model that best reproduce the participant's motion. Tracking errors for the different DOFs were below 1°. Using inverse dynamics, the joint moments were calculated for each participant. The residual reduction algorithm (RRA), which slightly adjusts the joint kinematics, trunk COM location and model mass properties, was then used to optimize the simulation's dynamic consistency. Using the adjusted model and kinematics determined from RRA, the computed muscle control algorithm (CMC) was used to generate a set of excitations of the torque actuators at the individual DOF (Thelen et al., 2003; Thelen and Anderson, 2006). These excitations produce a coordinated torque-driven simulation that accurately tracks the participant's movement. Induced acceleration analysis was then used to compute the contributions of individual actuators to the forward acceleration of the body COM at each time point of the participant's gait simulation, more specific the acceleration of the COM was evaluated for an instantaneous increase of the torque actuator with 1 Nm (Figure 1) (Zajac and Gordon, 1989; Riley and Kerrigan, 1999; Anderson and Pandy, 2003). For each actuator of the right and left limbs, we calculated the mean induced acceleration produced during loading response, single stance, preswing and swing separately. Likewise, the contribution of gravity was calculated. To test simulation accuracy, superposition was verified and the error was on average 0.22 m/s^2^ for horizontal acceleration varying between 2.37 and −2.43 m/s^2^. Maximal errors up to 0.78 m/s^2^ were reported, mainly at the instant of initial contact, a finding reported by others previously (Liu et al., 2006). Total induced acceleration was calculated for the actuators of the right and left upper and lower limbs separately. Finally, the percentage contribution of the actuators of the different segments (upper limbs, lower limbs) and gravity was expressed with respect to the combined total contribution of the segments and gravity. For HeCP, averages for affected and unaffected side were calculated separately. For TD and DiCP, values of right and left side were averaged for the respective gait phases.

Given the small sample size, individual data points, and descriptive statistics (mean and standard deviation) were used to describe the differences between the groups.

## Results

**Figure 2:**
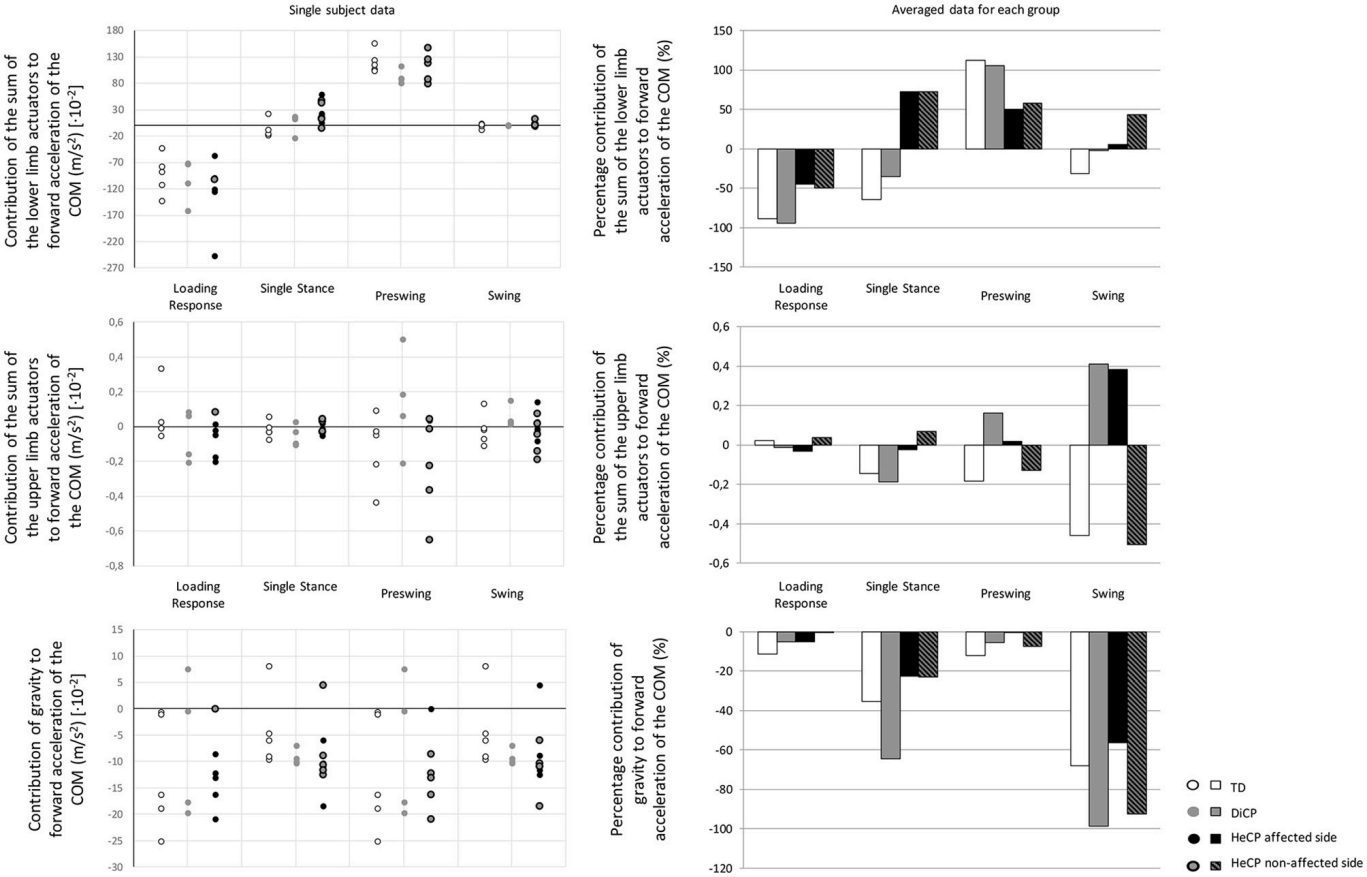
**Single subject data (left) and the averaged data for the three groups of the percentage contribution (right) to forward acceleration/deceleration of the COM (Typically Developing children [TD], children with diplegic cerebral palsy [DiCP], and children with hemiplegic cerebral palsy [HeCP]) for the lower limbs (upper graph), upper limbs (middle graph), and gravity (lower graph) at the different phases of the gait cycle (loading response, single stance, preswing, and swing)**. The percentage contribution of the sum of the actuators of the upper limbs, lower limbs or gravity is expressed with respect to the combined total contribution of the segments and gravity.

For the three groups, the torque actuators of the lower limbs contributed the most to the forward acceleration of the body COM, while the torque actuators of the upper limbs contributed minimally (less than 1%; Figure 2, Table 2). The magnitude of the contribution of the lower limbs and gravity were similar between the groups (Figure 2, Table 2). However, the contribution of the upper limbs was less consistent (Figure 2): In CP, the torque actuators of the upper limbs contributed more to forward acceleration of the COM during different phases in the gait cycle. In DiCP, the upper limbs seemed to contribute more to COM forward acceleration during preswing and swing. Similar results were found for the affected upper limb in HeCP. However, the unaffected arm in HeCP seemed to contribute more to COM forward acceleration during single stance.

**Table 2:** **Absolute average (***SD***) contribution to COM forward acceleration [·10^**−2**^]**.

	**Loading response**	**Single stance**	**Preswing**	**Swing**
**LOWER LIMBS**				
TD (m/s^2^)	–98.256 (43.334)	–10.624 (18.300)	117.708 (52.238)	–2.699 (10.482)
DiCP (m/s^2^)	–93.903 (36.685)	–5.241 (23.533)	98.874 (17.102)	–0.153 (1.037)
HeCP affected side (m/s^2^)	–127.231 (55.926)	31.077 (5.889)	81.462 (36.408)	–0.612 (1.679)
HeCP unaffected side (m/s^2^)	–102.566 (19.307)	18.109 (23.501)	112.312 (34.079)	–4.541 (6.809)
**UPPER LIMBS**				
TD (m/s^2^)	0.026 (0.178)	–0.024 (0.045)	–0.192 (0.292)	–0.039 (0.101)
DiCP (m/s^2^)	–0.011 (0.137)	–0.028 (0.062)	0.151 (0.235)	0.040 (0.058)
HeCP affected side (m/s^2^)	–0.088 (0.116)	–0.011 (0.062)	0.032 (0.123)	0.039 (0.099)
HeCP unaffected side (m/s^2^)	0.080 (0.116)	0.017 (0.088)	–0.253 (0.334)	–0.053 (0.097)
**GRAVITY**				
TD (m/s^2^)	–12.681 (11.713)	–5.849 (6.592)	–12.681 (11.713)	–5.8491 (6.592)
DiCP (m/s^2^)	–5.298 (11.479)	–9.565 (1.397)	–5.298 (11.479)	–9.565 (1.397)
HeCP affected side (m/s^2^)	–14.767 (4.699)	–9.742 (2.056)	–0.150 (0.874)	–5.720 (5.123)
HeCP unaffected side (m/s^2^)	–0.150 (0.874)	–5.720 (5.123)	–14.767 (4.699)	–9.742 (2.056)

*Table 2 presents the mean (SD) of the absolute values of the contribution to the forward Centre of Mass (COM) acceleration of the Lower Limbs, Upper Limbs and Gravity during Loading Response, Single Stance, Preswing, and Swing in the three groups (Typically Developing children [TD], children with diplegic cerebral palsy [DiCP], and children with hemiplegic cerebral palsy [HeCP])*.

### Timing between the upper and lower limbs' contributions

In TD the contribution of the lower limb actuators and upper limb actuators are synchronous during the loading response, single stance and swing phases of gait (Figure 2, Table 2). At these instances, both the upper and lower limb actuators decelerate the COM. Only during preswing, the lower limb actuators accelerate the COM while the upper limbs appear to decelerate the COM.

Contrarily, in DiCP the contribution of the lower limb and upper limb actuators are synchronous in all phases of gait (Figure 2, Table 2). All actuators decelerate the COM during the loading response and single stance, while they accelerate the COM during swing and preswing.

In HeCP, on the other hand, there is an asynchronous contribution of the upper and lower limb actuators during swing and preswing (Figure 2, Table 2). Similar as in TD, during preswing, the lower limb actuators accelerate while the upper limbs actuators decelerate the COM. During swing, the lower limb actuators in HeCP accelerate the COM while the upper limb actuators of the non-affected side decelerate the COM.”

## Discussion

The aim of the current study was to investigate whether upper limb movements influence forward acceleration of the COM during walking, in particular in hemiplegic and diplegic CP. The current results confirmed our hypothesis that there was minimal contribution of the upper limb muscles to propulsion of the COM in TD children. Even though the current findings seemed to confirm our hypothesis that the contribution to COM propulsion of the arms in DiCP and HeCP was altered compared to TD, the contribution of the upper limbs to the propulsion of the COM was also minimal in both CP groups compared to the contribution of the lower limbs.

Nevertheless, from the descriptive statistics it appeared that in DiCP, the upper limb contribution to the COM acceleration was increased during preswing and swing compared to TD, indicating that children with DiCP rely more on additional acceleration of the COM through arm swing during phases where propulsion is important.

In HeCP, strikingly, both the affected and unaffected upper limbs appear to compensate for the reduced contribution of the lower limbs; i.e., the unaffected side showed an increased contribution to COM acceleration during single stance, while the affected side contributes more to acceleration during preswing and swing.

Furthermore, it appeared that the timing between the contribution of the upper and lower limbs to the forward acceleration/deceleration is different compared to TD. In TD the contribution of the lower limb and upper limb actuators are synchronous during the loading response, single stance and swing phases of gait, and asynchronous during preswing. From the current results, it appeared that in DiCP, the contribution of the upper and lower limbs was synchronous in all phases of gait. In HeCP, however, we found that during swing, the lower limb actuators contribute to the forward acceleration of the COM while the upper limb actuators of the non-affected side contribute to the deceleration of the COM.

Even though the contribution of the arms to COM propulsion in DiCP and HeCP seemed altered compared to TD, their contribution to forward COM acceleration remains negligible compared to that of the lower limbs and gravity (similarly as in TD). Additionally, the timing of the contribution between the upper and lower limb actuators differ between TD and both CP groups. Combined this might suggest that the altered contribution of the upper limb movements to COM propulsion may be related to their coordination deficits (Meyns et al., 2012b), rather than a compensation strategy to increase forward acceleration of the COM. Hence, the clinical implication of the current study is that the arm movements do not need to be incorporated in the gait rehabilitation of children with CP to increase the propulsion of the COM. On the other hand, from the current results, it appears that the natural arm movements in children with CP should also not necessarily be discouraged, even though they are altered, as there does not appear to be a negative effect (i.e., increased deceleration) on the COM. On the other hand, the natural arm movements in children with CP have been related to gait stability (Meyns et al., 2012a, 2016). When the arms are not allowed to move during walking, children with CP show a decreased gait stability, especially in bilaterally affected children (Delabastita et al., 2016). Hence, from this point of view the natural arm movements in children with CP should not be discouraged or unlearned in gait rehabilitation. A next step in research could be to investigate the contribution of the upper limbs to medio-lateral acceleration of the COM as a measure of gait stability. Furthermore, future research could focus on the effect of balance training on gait stability in children with CP and whether this will induce changes in arm movements during walking in these children. Additionally, it is of interest to determine the effect of botulinum toxin treatment of spastic upper limb muscles on the arm movements during gait in children with CP and whether this will have an effect on their gait stability.

When interpreting the results of the current study, one should take into account some limitations. The sample size of the study is too small to perform statistical comparisons. Hence, this study provides inconclusive results concerning the differences on the effect of the upper and lower limb actuators on the forward COM acceleration between children with hemiplegia, diplegia and typically developing children. Nevertheless, from the descriptive results it is safe to state that the arms do not contribute significantly to linear accelerations of the COM in CP and typical gait. There are some limitations concerning the use of simulation techniques. The models used in this study were scaled from adult models and it is possible that they cannot account for the possible bone deformities or altered muscle physiology of the included children with cerebral palsy. The included participants did not show significant bone deformities. The use of such a simplified model could have affected the results to some degree. On the other hand, the difference in contribution between the lower limb actuators and upper limb actuators for each group was of such an extent that using another model will only show negligible changes.

## Ethics statement

The local ethical committee (Commissie Medische Ethiek KU Leuven [Medical Ethics Committee UZ KU Leuven/Research]) approved the experiments. In accordance with the Declaration of Helsinki, written informed consent was obtained of the participants' parents prior to the experiment. The participants parents supervised the measurement session.

## Author contributions

PM, GM, JD, and IJ conceived and designed the experiment. PM performed the experiments. PM and GM performed patient recruitment. PM and IJ analyzed the data. PM wrote the paper. The writing process and the data analysis were supervised by GM, JD, and IJ.

## Funding

PM was supported by grants of the Special Research Fund of the KU Leuven (OT/08/034 and PDMK/12/180), and by a grant of the FWO (grant G.0901.11). PM is a Marie Skłodowska-Curie fellow (proposal 660458). JD is recipient of a CNPq Visiting Professor Grant (400819/2013-9). This project was supported by the IWT-TBM (SimCP IWT-TBM project). There was no role of these funding bodies in the study design, collection and analysis of data, interpretation of data, writing of the paper, and decision to submit the paper for publication.

### Conflict of interest statement

The authors declare that the research was conducted in the absence of any commercial or financial relationships that could be construed as a potential conflict of interest. The handling Editor currently co-hosts a Research Topic and declares a shared affiliation with one of the authors PM, but confirms the absence of any other collaboration. She states that the process met the standards of a fair and objective review.
